# Breaking Away From the Male Stereotype of a Specialist: Gendered Language Affects Performance in a Thinking Task

**DOI:** 10.3389/fpsyg.2018.00985

**Published:** 2018-06-19

**Authors:** Marlene Kollmayer, Andreas Pfaffel, Barbara Schober, Laura Brandt

**Affiliations:** Department of Applied Psychology: Work, Education and Economy, Faculty of Psychology, University of Vienna, Vienna, Austria

**Keywords:** gender bias, gender-fair language, masculine generics, sex roles, stereotyping

## Abstract

This experimental online-survey study investigated if different written language forms in German have an effect on male bias in thinking. We used answers to the *specialist riddle* as an indicator for male bias in mental representations of expertise. The difficulty of this thinking task lies in the fact that a gender-unspecified specialist is often automatically assumed to be a man due to gender stereotypes. We expected that reading a text in gender-fair language before processing the specialist riddle helps readers achieve control over automatically activated gender stereotypes and thus facilitates the restructuring and reinterpretation of the problem, which is necessary to reach the conclusion that the specialist is a woman. We randomly assigned 517 native German speakers (68% women) to reading a text on expertise written either in gender-fair language or in masculine generics. Subsequently, participants were asked to solve the specialist riddle. The results show that reading a text in gender-fair language before processing the riddle led to higher rates of answers indicating that the specialist is a women compared to reading a text in masculine generics (44% vs. 33%) in women and men regardless of their self-stereotyping concerning agency and communion. The findings indicate that reading even a very short text in gender-fair language can help people break their gender-stereotype habit and thus reduce male bias in thinking. Our research emphasizes the importance of using gender-fair language in German-language texts for reducing gender stereotypes.

## Introduction

When reading, people form a mental model of the information in a text that contains representations of individuals and events relevant to the interpretation of the text (Garnham, 2001). In constructing this mental model, individuals rely on different types of background knowledge (Sanford and Garrod, 1981). One aspect of background knowledge people often unintentionally fall back on is stereotypical gender information (Carreiras et al., 1996). These automatically activated gender stereotypes can cause false inferences designated as gender bias. The following riddle (Stöger et al., 2004) illustrates how falling back on stereotypical gender knowledge causes such a bias:

A father and his son driving together in their car have a terrible car accident. The father dies upon impact. The son is rushed to the hospital in an ambulance and is immediately brought to the operating table. The doctor takes a quick look at him and says that a specialist is needed. The specialist comes, looks at the young man on the operating table and proclaims: “I cannot operate on him, he is my son.” How can this be?

Many people reading this text are confused and have difficulties solving the riddle (Stöger et al., 2004). Their difficulties arise from attributing gender values to the characters occurring in the story. In the case of *father* and *son* the gender values are explicit as fathers and sons are definitely male. However, many readers attribute male gender to the gender-unspecified *specialist* as well which leads to an inconsistency in their mental model of the riddle. They think that the specialist is the boy’s father although earlier in the riddle it is stated that the boy’s father died. Thus, these readers have difficulties finding the highly plausible solution of the riddle that the specialist is the young man’s mother. The aim of the present study was to examine if different factors influence whether individuals find this solution to the *specialist riddle*, namely gender-fair language, and participant gender and self-stereotyping regarding agency and communion.

### Gender Stereotypes and the Specialist Riddle

The reason why many readers assign male gender to the specialist lies in gender stereotypes. In general, stereotypes associate a category with traits that are assumed to be typical of members of that category (Dijksterhuis and van Knippenberg, 1996). Men and women are thought to differ in terms of achievement-oriented traits (*agency, competence*, or *instrumentality*) and in terms of social- and service-oriented traits (*communion, warmth*, or *expressivity*) (Fiske et al., 2002; Kite et al., 2008). Stöger et al. (2004) used the riddle above to demonstrate that for most people a specialist – i.e., a competent and successful person – is male. Indeed, in their study only 32% of participants stated that the specialist is a woman. This is in line with studies finding brilliance and genius to be associated much more with men than with women (Upson and Friedman, 2012; Bian et al., 2017). The social role name specialist seems to activate a male prototype, whereby the gender of the specialist, a characteristic that is important for solving the specialist riddle, is considered to be verified due to a *partial-match process* (see Kamas et al., 1996). For finding the highly plausible solution that the specialist is a woman, a radical restructuring and reinterpretation of the problem is necessary. Although people understand the riddle linguistically, and have, in principle, the necessary information for solving it, they overlook a discrepancy between the question posed and the stereotypical information activated from their memory. Sanford (1985) was the first to point this out using a slightly different version of this riddle – the *surgeon riddle* – which was applied in several studies in the English language area (Carreiras et al., 1996; Oakhill et al., 2005; Reynolds et al., 2006). The version of the riddle above closely parallels the version described by Sanford (1985) in which it is not a *specialist* but a *surgeon* who recognizes her son on the operating table.

Gender stereotypes are activated immediately when category names, such as *specialist* or *surgeon*, are read (Devine, 1989; Oakhill et al., 2005), which is illustrated by the specialist riddle and the surgeon riddle, respectively. The difficulties in solving the riddle reported by a significant proportion of participants in different studies indicate that once activated, stereotypical gender information is difficult to suppress (Carreiras et al., 1996; Stöger et al., 2004; Oakhill et al., 2005; Reynolds et al., 2006). However, there is evidence from research on implicit race bias that individuals can gain control over immediately activated and often unintentional stereotypes (Devine et al., 2000). Implicit race bias can be reduced through a combination of awareness, concern about the effects of the bias, and the application of certain bias-reducing strategies. A number of stereotype control strategies – including perspective-taking (Galinsky and Moskowitz, 2000) or counter-stereotypic mental imagery (Blair et al., 2001) – were identified as effective. However, these strategies require high internal motivation and are difficult to implement on a larger scale (Devine and Monteith, 1999; Devine et al., 2002). For reducing biases caused by stereotypes in the long run, it is important to find cues for control that help people break their stereotype habit.

### Gender-Fair Language as a Cue for Control

Systematically inserting cues for control into texts might be a promising way to a long-term reduction of implicit gender bias (Monteith et al., 2002), and gender-fair language might be a possibility to achieve this goal. In grammatical gender languages (e.g., German, French, Italian) every noun has a grammatical gender and the gender of personal nouns generally expresses the gender of the designated person (Sczesny et al., 2016). Occupations and roles are therefore referred to differently depending on the gender of the individual holding this occupation or role (e.g., German Spezialist/Spezialistin for [male/female] specialist). Nevertheless, it is a common practice in German that masculine role nouns generically serve as labels for mixed-gender groups or persons whose gender is unknown or unspecified. This practice has often been criticized by feminist linguists and psychologists (Stahlberg et al., 2007). In psychological research, numerous studies revealed that in German masculine generics evoke a male bias in mental representations even when their use is intended as neutral (Stahlberg and Sczesny, 2001; Sczesny et al., 2016). Moreover, several studies indicate that gender-fair language – sometimes also referred to as gender-neutral language (Sarrasin et al., 2012), gender-inclusive language (Stout and Dasgupta, 2011), gender-sensitive language (Savić, 2011), or non-sexist language (Douglas and Sutton, 2014) – leads to more cognitive involvement of women and can thus reduce or eliminate male bias in mental representations (e.g., Braun et al., 1998, 2005; Heise, 2000; Irmen and Linner, 2005; Stahlberg et al., 2007).

In grammatical gender languages such as German, two principle strategies are used to achieve gender-fairness, namely *neutralization* and *feminization*. Neutralization means replacing masculine forms (e.g., German Spezialist) with gender-neutral forms (e.g., German Koryphäe). However, this is not feasible for all masculine nouns. In contrast, feminization is based on explicitly including women in language. The inclusion of women can be achieved by either replacing masculine forms by feminine-masculine word pairs (e.g., German Spezialistinnen und Spezialisten for [female and male] specialists) or abbreviated forms with slashes (e.g., German Spezialist/in), brackets (e.g., German Spezialist[in]) or the so-called capital-I form (e.g., German SpezialistIn). Feminization is recommended to reduce the male bias in mental representations for grammatical gender languages (Hellinger and Bußmann, 2001; Moser et al., 2011). Besides the phonological and visual similarity to the feminine form, associations of feminized forms with political correctness and feminist ideas are assumed to increase the cognitive representation of women (Stahlberg and Sczesny, 2001). On the one hand, feminized forms produce a link between grammatical gender and gender of the designated person, leading to higher availability of female exemplars. On the other hand, they seem to initiate motivational processes that can help to overcome male biases.

A variety of dependent variables were used to demonstrate that gender-fair language increases the mental representation of female exemplars compared to masculine generics, for example estimations of the gender distribution in certain groups (Braun et al., 1998), sentence finishing tests (e.g., Rothmund and Scheele, 2004), recognition tasks (e.g., Rothermund, 1998), or reading and reaction times (e.g., Irmen and Köncke, 1996; Irmen and Roßberg, 2004). The effects of gender-fair language seem to be moderated by participants’ attitudes toward gender-fair language (Stahlberg and Sczesny, 2001).

### The Role of Gender and Self-Stereotyping

While participant gender does not seem to affect the male bias in thinking (Merritt and Kok, 1995), empirical evidence regarding the question whether participant gender moderates the effect of gender-fair language on the mental representation of women is inconclusive. Braun et al. (1998) found that women who read a text in gender-fair language reported a higher percentage of female representations than women who read the same text in masculine generics while in men the proportion of female representations was the same in both language conditions. In contrast, Stahlberg and Sczesny (2001) conducted three experiments in which they found both women and men naming more female exemplars if questions were formulated in gender-fair language than if questions were formulated in masculine generics. Not only studies examining gender differences regarding the effects of gender-fair language but also those examining gender differences in attitudes on gender-fair language are inconclusive. Although most studies found women to evaluate gender-fair language more positively than men (Rubin et al., 1994; Parks and Roberton, 2004; Braun et al., 2007; Douglas and Sutton, 2014), there are exceptions finding no such differences (Sczesny et al., 2015).

When examining gender differences in a non-essentialist way, it is crucial to consider not only the gender category (differences between men and women) but also the extent to which individuals identify with typical characteristics of masculinity and femininity, i.e., their self-stereotyping concerning agency and communion. Psychological masculinity is associated with achievement-oriented traits, labeled as agency, competence, or instrumentality, whereas psychological femininity is associated with social-oriented traits, designated as communion, warmth, or expressivity (Abele and Wojciszke, 2007; Kite et al., 2008; Donnelly and Twenge, 2017). Men and women can be high or low on both of the two independent dimensions. Bem (1974) argues, that self-stereotyping regarding agency and communion can over-ride participant gender regarding its impact on psychological functioning. Research shows that regardless of their identification with traditionally masculine and feminine characteristics individuals attribute male gender to a gender-unspecified character more often than female gender (Merritt and Kok, 1995). However, to our knowledge, there is no research investigating differential effects of gender-fair language on the mental representation of women with regard to self-stereotyping. Even research linking attitudes toward gender-fair language to self-stereotyping regarding agency and communion is sparse. Rubin et al. (1994) found that psychological masculinity is associated with less use of gender-fair language in writing tasks which indicates less positive attitudes toward gender-fair language in individuals strongly identifying with traditionally masculine characteristics.

### Present Study

Numerous studies have shown that the use of masculine generics restricts the cognitive availability of women (Braun et al., 1998; Heise, 2000; Irmen and Linner, 2005; Stahlberg et al., 2007). In the present study, we aimed to expand prior research by examining if reading a text in gender-fair language reduces gender bias in thinking as observed in individuals failing to break away from the male stereotype of a specialist when processing the *specialist riddle*. While previous studies investigating the effects of gender-fair language focused on gender bias in the sense of a reduced cognitive availability of female exemplars, using this riddle allowed examining whether people manage to break away from the male stereotype activated when reading the social role name *specialist*. From a cognitive perspective, breaking away from this stereotype requires a radical reinterpretation of the problem that seems to be difficult for many readers even after the original assumption that the specialist is a man has led to a contradiction.

Based on the assumption that gender-fair language increases the cognitive availability of female exemplars by cognitive and motivational processes (Stahlberg and Sczesny, 2001), we assumed that reading a text in gender-fair language facilitates the radical restructuring and reinterpretation of the problem, and thus helps readers to achieve control over automatically activated gender stereotypes. Specifically, we expected that reading a text on expertise in gender-fair language prior to processing the *specialist riddle* would result in higher rates of answers specifying that the specialist is a woman compared to reading the same text in masculine generics. By using answers to the specialist riddle as the dependent variable, we were able to examine the effect of gender-fair language not only on the cognitive availability of women, but also on stereotyping.

As participant gender does not seem to affect the male bias in thinking (Merritt and Kok, 1995), we expected men and women to provide similar rates of answers specifying that the specialist is a woman. Moreover, we examined differential effects of gender-fair language on gender bias in women and men, since prior studies led to inconsistent results (Braun et al., 1998; Stahlberg and Sczesny, 2001). As previous findings are equivocal, we had no *a priori* hypothesis regarding differential effects of gender-fair language on gender bias in women and men. Finally, we explored differential effects of gender-fair language on gender bias regarding readers’ self-stereotyping concerning agency and communion. Previous research indicates that individuals who strongly identify with stereotypically masculine characteristics have less positive attitudes toward gender-fair language which might influence the effects of gender-fair language (Rubin et al., 1994). To our knowledge, this is the first study on the effects of gender-fair language that included a measure of self-stereotyping, therefore, we had no *a priori* hypothesis.

## Materials and Methods

### Procedure

The present study was designed as an experimental online study. Participants were invited to complete the online questionnaire via social media and email, and also via online courses of the University of Vienna in order to reach university students and academics. Since it was not possible to reveal the actual topic of the investigation without distorting the results, participants were told that the study examined how individuals deal with riddles. An online consent form informed participants about duration and procedure of the study. Participants were guaranteed anonymity and confidentiality of their data, and were informed that participation was voluntary and could be withdrawn at any point of the questionnaire. After completing the informed consent form, participants answered an online questionnaire spanning demographics (gender, age, educational level, and native language), a randomly assigned priming text (in masculine generics or gender-fair language), the *specialist riddle*, and a measure assessing participants’ self-stereotyping regarding agency and communion.

### Materials

#### Language Condition: Gender-Fair Language vs. Masculine Generics

A priming text in masculine generics (language condition 1) or gender-fair language using the capital-I form (language condition 2) was the experimental manipulation in the present study. In Austria, equal linguistic treatment of women and men was first recommended in Wodak et al. (1987). Since then, different ministries, administrative bodies, public organizations, NGOs, and private enterprises developed guidelines for gender-fair language (Leitfäden und Literatur zum geschlechtergerechten Sprachgebrauch, 2012). While these guidelines propose different strategies to achieving equal linguistic representation of women and men, all of them introduce the capital-I as an acceptable form of gender-fair language. Although this form does not (yet) correspond to the spelling rules in German, the capital-I is found in numerous publications (Wetschanow, 2010). Therefore, the use of the capital-I is quite common in Austria, and it can be assumed that all study participants were familiar with this form. Participants were randomly assigned to reading one version of a short encyclopedia entry on expertise (100 words) that included seven terms referring to persons (underlined in the text below; not underlined in the study version). These terms were either masculine role nouns (language condition 1) or feminine role nouns with a capital-I (language condition 2). The priming text can be translated as follows (for the original German versions of the priming text see Supplementary Material):

In psychology, expertise or expert knowledge refers to exceptional problem solving skills or performance in a particular area that goes back to extensive experience. Outstanding experts are also known as specialists. Expertise is most often acquired in vocational training or studies, but can also be acquired through research or autodidactically. Research on expertise investigates the nature and acquisition of problem-relevant, area-specific knowledge. For this purpose, researchers usually compare the problem-solving behavior of experts and novices. In contrast to experts, novices are people who do not have the relevant experience in the corresponding area.

Participants were asked to read the text and to answer a question about the text (*What is usually compared in expert research?*) to assure that they had read the text.

#### Gender Bias: Specialist Riddle

On the next page of the online questionnaire, participants were asked to try to solve the *specialist riddle*, presented in the beginning of the article at hand (see Supplementary Material for the original German version). Of note, based on Stöger et al. (2004) the term translated as *specialist* was not the German word *Spezialist* but the German word *Koryphäe* (luminary), which is a grammatically feminine Greek loan word that does not have a masculine form. In accordance with Stöger et al. (2004) the solution of the riddle was sought as follows: *Can the problem be solved and explained in one sentence? If you are of this opinion, state your solution in a single sentence. Otherwise, write ‘no.’*

We assumed that both types of answers, those indicating that the specialist is a man and those deeming the riddle unsolvable, are related to gender stereotypes automatically activated through the social role name *specialist* (Devine, 1989; Stöger et al., 2004; Oakhill et al., 2005). In contrast to previous research on the specialist/surgeon riddle that categorized answers as *correct* or *incorrect*, we grouped participants’ answers into the two more incisive categories *answers indicating that the specialist is a woman* and *answers not indicating that the specialist is a woman*. This is due to the fact that there are alternative solutions to the riddle (biological father – stepfather, gay parents) that are not incorrect but nevertheless indicate that the possibility of a female specialist was not taken into account. The frequencies of the different types of answers by language condition can be found in **Table 1**. A sample answer for the category *supernatural answers* was “The spirit of the father came to the operating room.” Answers that did not fit one of the categories such as “The specialist is a priest” were coded as *other answers*. On the next page of the questionnaire, participants were asked to indicate if they had already known the *specialist riddle* before participating in the study.

**Table 1:** Absolute (relative) frequencies of participants’ answers to the *specialist riddle* by language condition.

Answers categorized	Language condition	
	Masculine generics (*n* = 197)	Gender-fair language (*n* = 192)
*Answers indicating that the specialist is a woman*		
Mother	64 (32.5%)	85 (44.3%)
Woman/female specialist	2 (1.0%)	0 (0.0%)
*Answers not indicating that the specialist is a woman*		
Riddle is unsolvable	53 (26.9%)	50 (26.0%)
Biological father (stepfather died)	53 (26.9%)	36 (18.8%)
Gay parents	11 (5.6%)	3 (1.6%)
Supernatural answers	3 (1.5%)	5 (2.6%)
Grandfather, father-in-law	1 (0.5%)	2 (1.0%)
Other answers	10 (5.1%)	11 (5.7%)

#### Self-Stereotyping: Bem Sex-Role Inventory

The last section of the questionnaire contained the German version of the Bem Sex-Role Inventory (BSRI) (Schneider-Düker and Kohler, 1988). Like the original BSRI, this version consists of 60 positively valued personality characteristics, that participants rate on a 7-point Likert scale as to how much they apply to them (never or almost never true – always or almost always true). It includes a *Masculinity scale* (sample items: *assertive, independent, and forceful*), a *Femininity scale* (sample items: *gentle, understanding, and warm*), and a gender-neutral *Social Desirability scale* (sample items: friendly, helpful, and sincere) with 20 items each. Each participant was given a *Masculinity score* and a *Femininity score*. For both scales, higher scores indicated more masculine or feminine self-stereotyping. Based on a median split, participants were classified as *masculine* (high masculinity and low femininity), *feminine* (low masculinity and high femininity), *androgynous* (high in both dimensions), or *undifferentiated* (low in both dimensions), as proposed by Spence et al. (1975).

### Participants

A total of 517 participants, 67.9% women, aged 17 to 66 years (*M* = 28.1 years, *SD* = 9.28) completed the online questionnaire. Only participants who had not known the *specialist riddle* before answering the questionnaire were included in the analyses. Thus, the final sample consisted of 389 participants, 68.4% of whom were women. A cross-tabulation of subjects’ self-stereotyping concerning agency and communion by gender can be found in **Table 2**. BSRI classifications were evenly distributed between both language conditions in men, χ^2^(3, 266) = 3.193, *p* = 0.36, and in women, χ^2^(3, 123) = 3.323, *p* = 0.35. The participants in the final sample reported their educational levels as follows: 0.5% had completed compulsory education, 3.3% vocational training, 67.4% high school, and 26.4% held a university degree. Half of the participants (*n* = 197, 50.6%) were randomly assigned to language condition 1 (masculine generics), and 192 participants (49.4%) were randomly assigned to language condition 2 (gender-fair language).

**Table 2:** Participants’ BSRI classification by gender.

		BSRI classification				
		
		Undifferentiated	Feminine	Masculine	Androgynous	Total
Gender	Men	38	13	44	28	123
	Women	63	86	48	69	266
	Total	101	99	92	97	389

## Results

The average rate of answers indicating that the specialist is a woman over both language conditions was 38.8% (*n* = 151). In language condition 1 (masculine generics) only 33.5% (*n* = 66) of the participants indicated that the specialist was a woman, whereas in language condition 2 (capital-I form) 44.3% (*n* = 85) of the participants’ answers indicated that the specialist was a woman. Because our *a priori* prediction was directional, we used a one-tailed test to analyze the effect of the experimental condition, Fisher’s exact test: *p* = 0.019 (one tail), Odds-Ratio = 1.58.

In order to analyze the effects of language condition, gender, self-stereotyping, and all interactions, we performed a stepwise (forward LR) binary logistic regression with answers indicating that the specialist is a woman vs. answers not indicating that the specialist is a woman as the dependent variable, and language condition, gender, BSRI classification, and all possible interactions as independent variables. We decided to use a stepwise method to test all predictors and interactions in one model in which variable entering is purely based on a statistical criterion, and overfitting by including non-significant predictors and interactions is avoided. The logistic regression model converged after two steps, which means that the model fit could not be significantly improved by including further independent variables.

**FIGURE 1:**
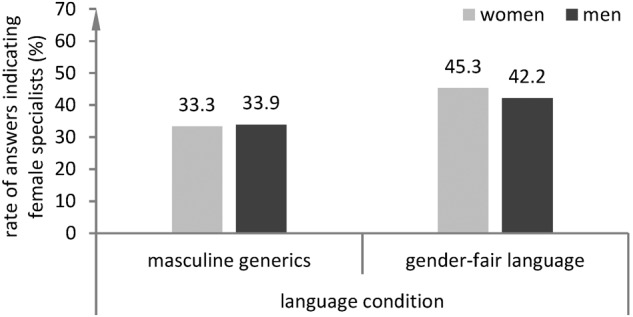
Rates of answers indicating that the specialist is a woman by language condition and gender.

**FIGURE 2:**
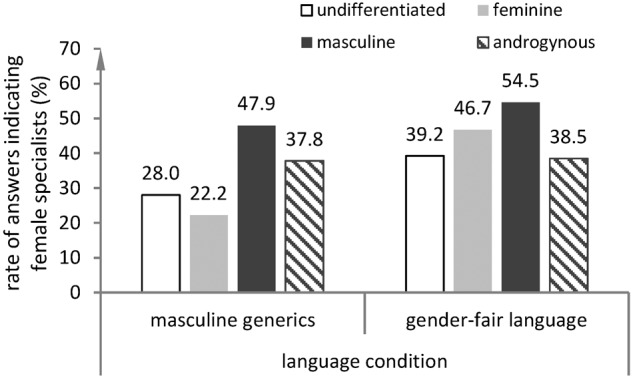
Rates of answers indicating that the specialist is a woman by language condition and BSRI classification.

The results of the regression model (**Table 3**) show a significant main effect of the language condition (*p* = 0.027, Odds-Ratio = 1.60) and a significant main effect of the BSRI classification (*p* = 0.041) but no significant effect of gender and no significant effect of any interactions. The results indicate that reading a text in gender-fair language led to higher rates of answers indicating that the specialist is a woman both in men and in women, see also **Figure 1**. The significant main effect of the BSRI classification indicates that at least one of the four groups differed significantly from other groups in their rate of answers indicating that the specialist is a woman, independent of the language condition (**Figure 2**). In detail, the results show that participants classified as *masculine* provided answers indicating that the specialist is a woman more often than all other groups in both language conditions, *p* = 0.013, Odds-Ratio = 2.10. Descriptive results indicate that while participants classified as androgynous or masculine showed very similar rates of answers indicating that the specialist is a woman in both language conditions, participants classified as feminine or undifferentiated provided answers indicating that the specialist is a woman noticeably more often in the gender-fair language condition than in the masculine generics condition (see **Figure 2**). However, this descriptive result should be interpreted with caution as the interaction of self-stereotyping and language condition was not significant in our regression model.

**Table 3:** Results of the stepwise logistic regression model predicting answers indicating that the specialist is a woman.

	*b* (*SE*)	Wald-χ^2^	*df*	*p*	OR
*Step 1 (included)*		
Language condition	0.455 (0.210)	4.724	1	0.030	1.58
Constant	–0.686 (0.151)	20.626	1	<0.001	
*Step 2 (included)*		
Language condition	0.469 (0.212)	4.885	1	0.027	1.60
BSRI classification^a^		8.266	3	0.041	
Feminine	0.009 (0.302)	0.001	1	0.977	1.01
Masculine	0.744 (0.299)	6.207	1	0.013	2.10
Androgynous	0.183 (0.299)	0.374	1	0.541	1.20
Constant	–0.691 (0.153)	20.414	1	<0.001	
*Step 2 (excluded)*		
Gender		0.740	1	0.390	
Language cond. ^∗^ gender		1.121	1	0.290	
Language cond. ^∗^ GRO		3.528	3	0.317	
Gender ^∗^ GRO		2.543	3	0.468	
Language cond. ^∗^ gender^∗^GRO		2.200	3	0.532	

*OR, Odds-Ratio; ^*a*^BSRI classification ‘undifferentiated’ as baseline; Model parameters of step 2: *R*^*2*^ = 0.03 (Cox & Snell), 0.05 (Nagelkerke); -2 Log L = 506.6; *p* = 0.011.*

## Discussion

The aim of the present study was to investigate if written gender-fair language reduces readers’ gender bias in mental representations of stereotypically masculine roles. Therefore, we conducted an experimental study, in which participants were randomly assigned to reading the same text on expertise either in gender-fair language or in masculine generics. In the gender-fair language condition, we used the form of feminization that was found to lead to the highest percentage of female representations – the capital-I form (Stahlberg and Sczesny, 2001). We chose the *specialist riddle* as our dependent variable as numerous previous studies have used this riddle to demonstrate that gender stereotypes are activated automatically and often unintentionally from social role names which leads to difficulties in finding the highly plausible solution that the specialist is a woman (Garnham et al., 2002; Oakhill et al., 2005; Reynolds et al., 2006). Using this riddle allowed us to examine whether gender-fair language enables people to break away from the male stereotype activated when reading the social role name *specialist*.

Our results show that the majority of subjects indeed had difficulties breaking away from the male stereotype of a specialist, despite the use of a grammatically feminine replacement for the noun specialist in the German version of the riddle. In the whole sample, more than 60% of participants did not provide an answer indicating that the specialist is a woman. The rate of answers indicating that the specialist is a woman in our study is consistent with the solution rates reported by Stöger et al. (2004). This demonstrates that a specialist is still assumed to be a man, which is in line with traditional gender stereotypes, characterizing men as agentic, competent, independent, and decisive, and women as emotional, kind, understanding, and concerned about others (Kite et al., 2008).

The main aim of our study was to investigate if gender-fair language can reduce gender bias in mental representations of specialists. Therefore, we examined if the rate of answers indicating that the specialist is a woman varied depending on the language (gender-fair language vs. masculine generics) used in a short priming text participants were randomly assigned to read before processing the *specialist riddle*. We found significant differences in these rates depending on the language condition. Only 33.5% of participants who had read a text on expertise in masculine generics provided an answer indicating that the specialist is a woman, while 44.3% of participants who read the same text (with regard to content) in gender-fair language indicated that the specialist was a woman. Gender-fair language seems to reduce the effect of the immediate activation of gender stereotypes from the stereotypically masculine social role name *specialist* and to facilitate the radical restructuring and reinterpretation of the problem necessary for solving the riddle and to reach the conclusion that the specialist is a woman, respectively. This might be due to the capital-I’s phonological and visual similarity to the feminine form, and also due to the association of the capital-I form with feminist ideas. Therefore, gender-fair language can be seen as a cue for control that helps people break their stereotype habit. As our design does not allow a causal interpretation, the results could also be interpreted as such that it is not gender-fair language that reduces the male bias but rather masculine generics that create the male bias in the first place (Silveira, 1980; Hamilton, 1988). Nevertheless, it is a common practice in German to use masculine generics as labels for mixed-gender groups or persons whose gender is unknown or unspecified, and sometimes even for women.

We also examined differential effects of gender-fair language on gender bias in men and women. Rates of answers indicating that the specialist is a woman within the two language conditions did not differ by gender. Both women and men who had read a text in gender-fair language before processing the riddle provided answers indicating that the specialist is a woman more often than women and men who had read the same text in masculine generics. This is in line with findings indicating that gender-fair language increases the availability of female exemplars both in men and women compared to masculine generics (Stahlberg and Sczesny, 2001). Stöger et al. (2004) used a newspaper report about a successful female/male scientist as a prime before presenting the specialist riddle. Interestingly, their results show that reading about successful female scientists leads to higher solution rates of the specialist riddle in women but not in men. This indicates that in men gender-fair language might have a stronger impact on the cognitive availability of women than a concrete reference to a woman in a non-stereotypical role. This might be related to feminized language forms being associated with political correctness and feminist ideas (Stahlberg and Sczesny, 2001) and thereby functioning as a cue for control against automatically activated gender stereotypes.

In addition, we examined differential effects of gender-fair language depending on how much participants identify with stereotypical masculine and feminine characteristics. We found that reading a text in gender-fair language led to higher rates of answers indicating that the specialist is a woman than reading a text in masculine generics regardless of participant self-stereotyping regarding agency and communion. However, individuals classified as masculine provided more answers indicating that the specialist is a woman than all other individuals in both language conditions. Considering that the majority of participants were women, this finding may be explained by a lower internalization of gender stereotypes in women who identify most with stereotypical masculine qualities. It is plausible that women who internalized gender stereotypes to a lesser extent managed to break away from the male stereotype of a specialist easier than women who internalized gender stereotypes to a greater extent. We found no statistically significant differential effects of gender-fair language on gender bias in mental representations of expertise depending on how much participants identified with traditionally masculine or feminine qualities. However, among participants classified as feminine the rate of answers indicating that the specialist is a woman was more than twice as high when they had read a text in gender-fair language than when they had read the same text in masculine generics while rates of answers indicating that the specialist is a woman among androgynous participants were almost the same in both language conditions. Given the partly small cell frequencies (see Supplementary Table S1 of the frequencies of solutions indicating that the specialist is a woman by gender and self-stereotyping in both language conditions), these descriptive results have to be interpreted with caution. However, they may indicate that individuals identifying with traditional feminine characteristics potentially benefit most from the use of gender-fair language, which should be examined by future research.

### Practice Implications

Although the effects of gender-fair language on gender bias in mental representations are small in size, the results are promising in the light of the very low threshold intervention realized in this experimental study. The priming text that was presented either in masculine generics or in gender-fair language was very short (100 words) and contained only seven terms referring to persons. Since the 1980s, several guidelines for gender-fair language were published with the purpose of achieving a fair treatment of women and men in language (for an overview see Moser et al., 2011). However, these guidelines are not prescriptive and mainly implemented in universities and public service while there are many areas in which gender-fair language is scarce. For example, Moser and Masterson (2014) found a preponderance of male terms in children’s books which might contribute to the development of gender stereotypical ideas in childhood. Moreover, Moser and Hannover (2014) found that schoolbooks for German contained more gender fair terms than schoolbooks for mathematics which might contribute to gender-stereotypical educational and occupational careers (Kollmayer et al., 2016). Considering that implicit biases are seen as major contributors to the perpetuation of discrimination (Devine et al., 2012) and that even very short texts have the potential to reduce male bias in mental representations, it seems necessary to implement gender-fair language on a larger scale, especially in stereotypically masculine contexts and domains, to promote efforts in ending discrimination.

### Limitations and Future Research

Three limitations of the present study should be mentioned. First, although the specialist riddle is an established tool for assessing gender bias, its major disadvantage is the dichotomous outcome (answers indicating that the specialist is a woman vs. answers not indicating that the specialist is a woman). This dichotomy only allows determining whether participants stuck to their stereotype inferences but not investigating fine gradations in implicit attitudes, as do measures such as the Implicit Association Test (IAT) (Greenwald et al., 1998). Therefore, the exact processes by which gender-fair language reduces gender bias remain unclear. One possibility is that gender-fair language leads to less automatic activation of gender stereotypes in the first place, and thus to higher rates of answers indicating that the specialist is a woman. The second possibility is that gender-fair language did not influence the automatic activation of gender stereotypes but worked as a cue for control that subsequently helped participants gain control over the immediately activated gender stereotypes (Devine et al., 2000; Monteith et al., 2002). Quantitative methods from neuropsychology could contribute to a deeper understanding of the processes of stereotype activation (Ferstl and Kaiser, 2013). A second limitation lies in the small cell frequencies resulting from combining the two language conditions with gender and the four BSRI classifications (see Supplementary Table S1). As descriptive results indicate complex interactions, further studies with larger sample sizes could examine if women or men identifying with traditional masculine and feminine characteristics to a certain degree benefit more from the use of gender-fair language than others. A third limitation of the present study is that we did not include a measure of attitudes toward gender-fair language in our study although Stahlberg and Sczesny (2001) found negative attitudes toward gender-fair language to reduce the positive effects of gender-fair language on the cognitive inclusion of women. However, due to the random assignment of participants we assume attitudes toward gender-fair language to be equally distributed in both language conditions.

## Conclusion

The present study contributes to a better understanding of the role masculine generics play in the perpetuation of gender stereotypes. Using a riddle difficult to solve for people who assign male gender to a person introduced as a specialist, we demonstrated that reading a text in gender-fair language reduces gender bias in mental representations of expertise compared to reading the same text in masculine generics. This indicates that the often-observed automatic gender stereotyping does not equate inevitable gender stereotyping. However, the social convention of using masculine nouns as generic seems to make it difficult to think of female exemplars, and thus reinforces gender bias in mental representations. In contrast, gender-fair language seems to be a promising cue for control to help people break their stereotype habit.

## Ethics Statement

The study was carried out in compliance with ethical standards of the Austrian Federal Ministry of Health (Bundesministerium für Gesundheit, 1995) and the American Psychological Association (American Psychological Association, 2010). Prior to participation, students gave written informed consent. The consent form informed participants about duration and procedure of the study. Participants were guaranteed anonymity and confidentiality of their data and were informed that participation was voluntary and could be withdrawn at any point of the questionnaire. According to Austrian and European (EU) law, approval of an ethics committee was not necessary as this study did not involve patients, was non-invasive, and participation was voluntary and anonymous. At the time of study conduct, there was no institutional review board (IRB) at the University of Vienna, where this research was conducted. Hence, no IRB approval was necessary.

## Author Contributions

All listed authors contributed meaningfully to the paper. MK and BS developed the study concept. All authors contributed to the study design, analyzed, or interpreted the data. MK and AP prepared the draft manuscript and LB provided critical revisions. All authors approved the final version of the manuscript for submission.

## Conflict of Interest Statement

The authors declare that the research was conducted in the absence of any commercial or financial relationships that could be construed as a potential conflict of interest.
