# Supplementary material for: Breaking Away From the Male Stereotype of a Specialist: Gendered Language Affects Performance in a Thinking Task
Source: Front Psychol. 2018 Jun 19;9:985. doi: 10.3389/fpsyg.2018.00985 (PMC6018092; doi:10.3389/fpsyg.2018.00985)
Supplement: Supplementary file 1 [file Table_1.PDF]

Table S1. Absolute frequencies of answers indicating or not indicating that the specialist is a woman by language condition, and participants' gender and gender-role orientation.

language condition	gender	BSRI classsification	answers indicating that the specialist is a woman	answers not indicating that the specialist is a woman	total
masculine generics	women	undifferentiated	23	8	31
		feminine	35	10	45
		masculine	14	16	30
		androgynous	20	12	32
		total	92	46	138
	men	undifferentiated	13	6	19
		feminine	7	2	9
		masculine	11	7	18
		androgynous	8	5	13
		total	39	20	59
	total		131	66	197
gender-fair language	women	undifferentiated	19	13	32
		feminine	22	19	41
		masculine	6	12	18
		androgynous	23	14	37
		total	70	58	128
	men	undifferentiated	12	7	19
		feminine	2	2	4
		masculine	14	12	26
		androgynous	9	6	15
		total	37	27	64
	total		107	85	192
total			238	151	389
