# Supplementary material for: Breaking Away From the Male Stereotype of a Specialist: Gendered Language Affects Performance in a Thinking Task
Source: Front Psychol. 2018 Jun 19;9:985. doi: 10.3389/fpsyg.2018.00985 (PMC6018092; doi:10.3389/fpsyg.2018.00985)
Supplement: Supplementary file 2 [file Presentation_1.PDF]

Breaking away from the male stereotype of a specialist: Gendered language affects performance in a thinking task

Marlene Kollmayer*, Andreas Pfaffel, Barbara Schober, Laura Brandt

*** Correspondence:** Marlene Kollmayer, marlene.kollmayer@univie.ac.at

1 Original research instruments (in German)

1.1 Priming Text

Language condition 1: Masculine Generics

Bitte lesen Sie folgenden kurzen Text über Expertise und beantworten Sie im Anschluss die dazu gestellte Frage:

In der Psychologie bezeichnet Expertise oder Expertenwissen eine außergewöhnliche Problemlösefähigkeit oder Leistung in einem bestimmten Bereich, die auf umfassende Erfahrung zurückgeht. Herausragende Experten werden auch als Koryphäen bezeichnet. Expertenwissen eignet sich die Person in der Regel durch eine Ausbildung oder ein Studium an, es kann jedoch auch durch Forschung oder autodidaktisch erworben werden.

Expertiseforschung untersucht die Art und den Erwerb problemrelevanten, bereichsspezifischen Wissens. Hierzu wird meistens das Problemlöseverhalten von Experten und Novizen verglichen. Novizen sind im Gegensatz zu Experten Personen, denen die entsprechende Übung im betreffenden Inhaltsbereich fehlt. Untersuchte Wissensgebiete sind unter anderem Computerprogrammierung, Physik, Musik, Sport und Medizin.

Was wird in der Expertiseforschung meist verglichen?

Language condition 2: Gender-fair language (capital-I form)

Bitte lesen Sie folgenden kurzen Text über Expertise und beantworten Sie im Anschluss die dazu gestellte Frage:

In der Psychologie bezeichnet Expertise oder ExpertInnenwissen eine außergewöhnliche Problemlösefähigkeit oder Leistung in einem bestimmten Bereich, die auf umfassende Erfahrung zurückgeht. Herausragende ExpertInnen werden auch als Koryphäen bezeichnet. ExpertInnenwissen eignet sich die Person in der Regel durch eine Ausbildung oder ein Studium an, es kann jedoch auch durch Forschung oder autodidaktisch erworben werden.

Expertiseforschung untersucht die Art und den Erwerb problemrelevanten, bereichsspezifischen Wissens. Hierzu wird meistens das Problemlöseverhalten von ExpertInnen und NovizInnen verglichen. NovizInnen sind im Gegensatz zu ExpertInnen Personen, denen die entsprechende Übung im betreffenden Inhaltsbereich fehlt.

Was wird in der Expertiseforschung meist verglichen?

1.2 The specialist riddle

Ein Vater und sein Sohn fahren gemeinsam im Auto und haben einen grässlichen Autounfall. Der Vater ist sofort tot. Der Sohn wird mit Blaulicht ins Krankenhaus gefahren und sofort in den Operationssaal gebracht. Der Arzt besieht ihn sich kurz und meint, man müsse eine Koryphäe zu Rate ziehen. Diese kommt, sieht den jungen Mann auf dem Operationstisch und meint: "Ich kann ihn nicht operieren, er ist mein Sohn." Wie ist das möglich?

Ist das Problem lösbar und mit einem einzigen Satz begründbar? Falls Sie dieser Meinung sind, begründen Sie ihre Lösung in einem einzigen Satz. Ansonsten schreiben Sie "nein".
